# Supplementary material for: pH-Responsive Lipid Nanoparticles Achieve Efficient mRNA Transfection in Brain Capillary Endothelial Cells
Source: Pharmaceutics. 2022 Jul 27;14(8):1560. doi: 10.3390/pharmaceutics14081560 (PMC9413996; doi:10.3390/pharmaceutics14081560)
Supplement: Supplementary file 1 [file pharmaceutics-14-01560-s001.zip › 220608 Revised SI.pdf]

Supplemental Information

pH-responsive lipid nanoparticles achieve efficient mRNA transfection in brain capillary endothelial cells

Yu Sakurai [1,*,†], Himeka Watanabe [1,†], Kazuma Nishio [1,†], Kohei Hashimoto [1,†], Atsuki Harada [1,†], Masaki Gomi [2], Masayoshi Suzuki [1], Ryotaro Oyama [2], Takumi Handa [1], Risa Sato [1], Hina Takeuchi [1], Ryoga Taira [1], Kenta Tezuka [1], Kota Tange [3], Yuta Nakai [3],Hidetaka Akita [1,*] and Yasuo Uchida [1,*]

[1] Laboratory of DDS Design and Drug Disposition, Graduate School of Pharmaceutical Sciences, Tohoku University, 6-3 Aoba, Aramaki, Aoba-ku, Sendai, 980-8578, Japan; himeka.watanabe.q7@dc.tohoku.ac.jp (H.W.); kazuma.nishio.t7@dc.tohoku.ac.jp (K.N.); kohei.hashimoto.t7@dc.tohoku.ac.jp (K.H.); atsuki.harada.s3@dc.tohoku.ac.jp (A.H.); gomi.masaki.t6@dc.tohoku.ac.jp (M.G.); masayoshi.suzuki.s8@dc.tohoku.ac.jp (M.S.); oyama.ryotaro.p3@dc.tohoku.ac.jp (R.O.); takumi.handa.q2@dc.tohoku.ac.jp (T.H.); risa.sato.t8@dc.tohoku.ac.jp (R.S.); hina.takeuchi.t5@dc.tohoku.ac.jp (H.T.); ryoga.taira.q2@dc.tohoku.ac.jp (R.T.); kenta.tezuka.r5@dc.tohoku.ac.jp (K.T.)

[2] Laboratory of DDS Design and Drug Disposition, Graduate School of Pharmaceutical Sciences, Chiba University, 1-8-1 Inohana, Chuo-ku, Chiba, 260-0856, Japan

[3] DDS Research Laboratory, NOF CORPORATION, 3-3 Chidori-cho, Kawasaki-ku, Kawasaki, 210-0865, Japan; kota_tange@nof.co.jp (K.T.); yuta_nakai@nof.co.jp (Y.N.)

* Correspondence: yu.sakurai.e7@tohoku.ac.jp (Y.S.); hidetaka.akita.a4@tohoku.ac.jp (H.A.); yasuo.uchida.c8@tohoku.ac.jp (Y.U.)

Tel.: +81-22-795-6833 (Y.S.); +81-22-795-6831 (H.A.); +81-22-795-6832 (Y.U.)

† These authors contributed equally to this work.

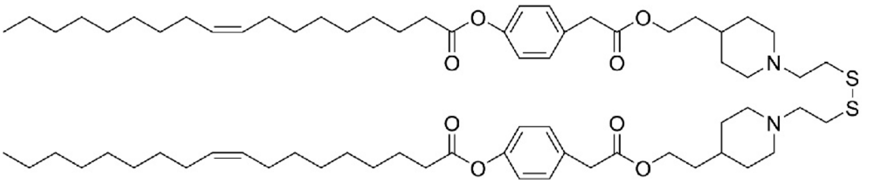

COATSOME™ SS-OP

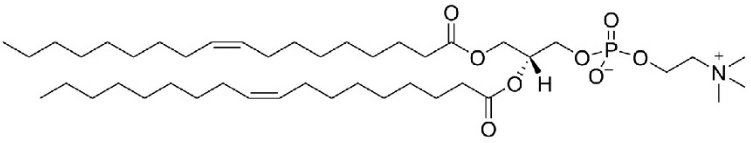

1,2-dioleoyl-*sn*-glycerophosphocholine (DOPC)

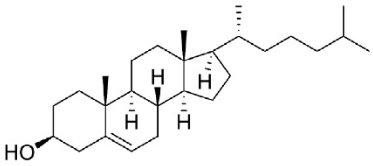

cholesterol (Chol)

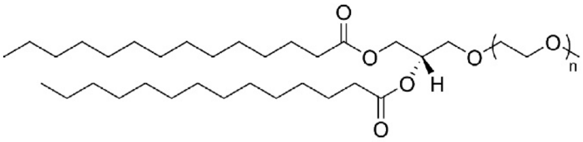

poly(ethylene) glycol (average molecular weight 2000)-1,2-dimyrisotyl-sn-glycerol (PEG-DMG)

Figure S1 The structures of the lipid molecules used in preparing the ssPalm LNP

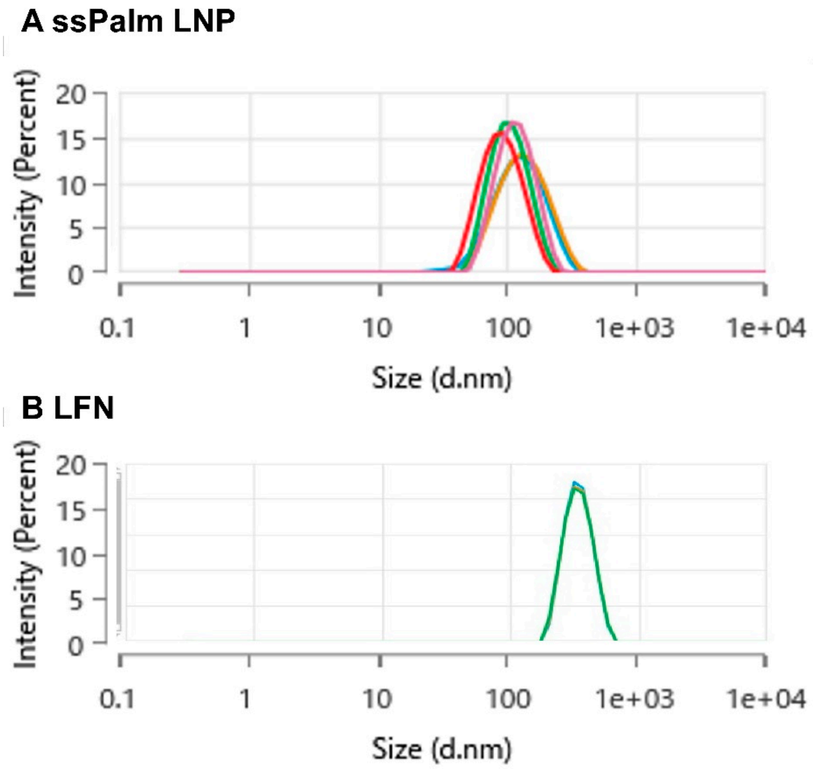

Figure S2 The particle size distribution of the ssPalm LNP and LFN. A,B, In the Figure, the size distributions of (A) ssPalm LNPs and (B) LFN independently prepared 5 or 3 times are plotted. Y-axis indicates intensity-based diameter of nanoparticles.

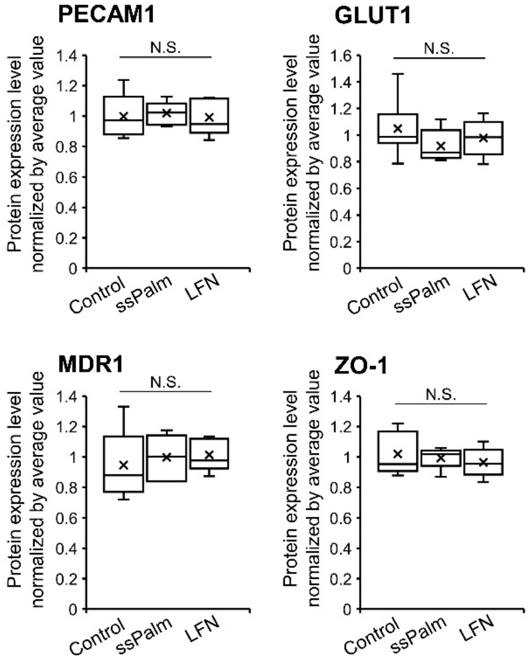

Figure S3 Protein expression levels of BBB marker proteins in hCMEC/D3 cells that had been treated with the ssPalm LNP and Lipofectamine. The ssPalm LNP containing GFP mRNA, and the complex of Lipofectamine MessengerMAX and GFP mRNA were prepared by the same procedure as the GFP transfection experiment, treated with hCMEC/D3 cells for 48 hours at a concentration of 2.5 μg/mL. A SWATH-MS analysis was then performed for the whole cell lysate of hCMEC/D3 cells. The protein expression levels of BBB marker proteins (PECAM1, GLUT1, MDR1, and ZO-1) were compared among the control, ssPalm and lipofectamine (LFN) groups (n = 5-9). The band inside the box represents the median, and the bottom and top of the box indicate the first and third quartiles, respectively. Whiskers indicate the minimum and maximum values of the protein levels. X plots show the average in each group. N.S., not significantly different among three groups (Benjamini–Hochberg adjusted p value > 0.05).
